# Integrating Health Behavior Theory and Design Elements in Serious Games

**DOI:** 10.2196/mental.4133

**Published:** 2015-04-21

**Authors:** Colleen Cheek, Theresa Fleming, Mathijs FG Lucassen, Heather Bridgman, Karolina Stasiak, Matthew Shepherd, Peter Orpin

**Affiliations:** ^1^ Rural Clinical School School of Medicine University of Tasmania Burnie Australia; ^2^ Werry Centre for Child and Adolescent Mental health Department of Psychological Medicine University of Auckland Auckland New Zealand; ^3^ Centre for Rural Health University of Tasmania Launceston Australia; ^4^ School of Counselling Human Services and Social Work University of Auckland Auckland New Zealand

**Keywords:** depression, adolescent, cognitive behavior therapy, online intervention, user-centered, self-efficacy, motivation, adherence, SPARX

## Abstract

**Background:**

Internet interventions for improving health and well-being have the potential to reach many people and fill gaps in service provision. Serious gaming interfaces provide opportunities to optimize user adherence and impact. Health interventions based in theory and evidence and tailored to psychological constructs have been found to be more effective to promote behavior change. Defining the design elements which engage users and help them to meet their goals can contribute to better informed serious games.

**Objective:**

To elucidate design elements important in SPARX, a serious game for adolescents with depression, from a user-centered perspective.

**Methods:**

We proposed a model based on an established theory of health behavior change and practical features of serious game design to organize ideas and rationale. We analyzed data from 5 studies comprising a total of 22 focus groups and 66 semistructured interviews conducted with youth and families in New Zealand and Australia who had viewed or used SPARX. User perceptions of the game were applied to this framework.

**Results:**

A coherent framework was established using the three constructs of self-determination theory (SDT), autonomy, competence, and relatedness, to organize user perceptions and design elements within four areas important in design: computer game, accessibility, working alliance, and learning in immersion. User perceptions mapped well to the framework, which may assist developers in understanding the context of user needs. By mapping these elements against the constructs of SDT, we were able to propose a sound theoretical base for the model.

**Conclusions:**

This study’s method allowed for the articulation of design elements in a serious game from a user-centered perspective within a coherent overarching framework. The framework can be used to deliberately incorporate serious game design elements that support a user’s sense of autonomy, competence, and relatedness, key constructs which have been found to mediate motivation at all stages of the change process. The resulting model introduces promising avenues for future exploration. Involving users in program design remains an imperative if serious games are to be fit for purpose.

## Introduction

### Background

Mental health conditions account for 13% of the global disease burden, with depression being the largest single cause of disability worldwide [1]. The large gap between treatment need and service provision is a global issue: in high income countries, 35% to 50% of people receive no treatment for severe mental disorders and in low income countries this fraction is much greater, with 76% to 85% not getting treatment [1]. The investment needed to provide trained therapists to fill these gaps is unfeasible [2], and not all potential patients favor or can access existing modes of treatment delivery. Integrating a range of accessible user-driven options into general community-level settings is one of the strategies promoted in the World Health Organization Mental Health Action Plan 2013-2020 [1]. There is an emphasis on early intervention, respecting the autonomy of individuals with mental health issues, and nonpharmacological therapies promoted particularly for young people [1].

The ubiquity of the Internet provides an opportunity for online computerized tools to extend the reach of psychotherapies such as cognitive behavioral therapy (CBT). While computerized therapies have been shown to be effective in alleviating depression and anxiety symptoms in adults, adolescents, and children [3,4], issues with user engagement and high attrition are noted in efficacy studies of the currently available text-based computerized cognitive behavioral therapy (cCBT) programs [5]. The opportunity for a more interactive and graphically rich experience via computer-based gaming technology has fueled development of serious games for mental health.

Gaming strategies that increase positive outcomes for mental health issues are only beginning to be explored; nevertheless, there have been a number of serious games developed to enhance mental well-being. These range from publically available but not necessarily clinically tested tools, such as Depression Quest, to programs which have been evaluated and reported in the peer-reviewed literature, such as gNats Island [6], SPARX [7], Camp Cope-A-Lot [8], Reach Out Central [9], and Virtual Iraq [10]. Most serious games for mental health issues that have been clinically tested are not publically available, and few describe the features of the game in any depth [11]. The gaming elements differ markedly among these: serious games for mental health include exercise programs with biofeedback, virtual reality simulations, word or number puzzles, and fantasy adventures. The program delivery processes also differ; some serious games (such as gNats Island) require a facilitator or clinician while others (such as SPARX) can be completed independently.

### Bringing Health Behavior Theory to Game Design

A model which incorporates elements that promote engagement and adherence and help users meet their goals could inform development and evaluation of serious games. Theories based on existing knowledge can provide a coherent framework to organize ideas and rationale clearly, facilitating communication among stakeholders [12]. Health interventions to promote behavior change that are based in theory and evidence and tailored to psychological constructs have been found to contribute to increased adherence and effectiveness [13]. In this instance, the psychological constructs of most interest are those that help explain which features of a serious game will support the individual to engage with the program, maintain interest in completing the tasks, and incorporate therapeutic concepts into everyday life.

Appealing to a user’s sense of self or agency and connectedness with others has been suggested to improve uptake and support engagement of computer-delivered therapies for depression and/or anxiety [14,15]. User-centered healthcare has gained momentum over recent decades and is very relevant with the expansion of consumer-based online resources and health technologies. More patient-led approaches to delivering services have also been recognized as a means of directing more efficient and effective use of health budgets [16]. Delivering health care in the 21st century requires personal access to tools that empower and allow citizens to pursue the best health strategies for themselves and their families [17]. This compels us to consider within the development process: “How can researchers construct participative health environments to support a patient’s sense of autonomy (personal control over health decisions), competency (mastery over self-management skills), and connectedness (social support from relevant others)—all factors implicated by psychological research to influence the intrinsic motivation of individuals? [18]”

These are the tenets of self-determination theory (SDT)[19]. Self-determination is defined by Deci and Ryan [20] as “a quality of human functioning that involves the experience of choice;” the options to choose are the determinants of an individual’s actions. SDT and other theoretical models of health behavior change, such as social-cognitive theory [21] and protection motivation theory [22], are considered continuum models; they predict that fulfillment of important psychological constructs will move an individual along a continuum toward behavior change and increase the likelihood of sustained outcomes. While continuum models are considered useful for explanation and prediction, stage models reflect the relative importance of different constructs at different stages of the behavior change process. From precontemplation through intention and action, interventions more specifically targeted to the needs of individuals at these different stages of health behavior change are considered more likely to improve recruitment, retention, and progress [23]. Stage theories of behavior change include the transtheoretical model [24] and the health action process approach (HAPA)[25]. HAPA demonstrates how understanding and designing to motivate users to access help, adhere to therapy, and sustain behavior change are critical in developing effective health interventions and include post-intentional volitional processes that lead to behavioral change [23]. In this model, self-efficacy and social support are important mediating factors at each stage of change, from contemplation through to actioning and sustaining change. Self-efficacy is defined as the confidence individuals must have in their ability to perform the desired action [25].

To provide a tool that can easily be communicated across stakeholder groups involved in serious game development, user perceptions based in psychological constructs need to be translated into practical design features that enable or support the desired user perceptions. Due to its relative immaturity as a formal discipline, the underlying theories and elements of design for serious games for mental health issues must be inferred from each of the stakeholder groups involved in development. Marne and colleagues [26] described the creation of serious games as a collaboration of two broad stakeholder groups: pedagogical experts and game experts. Each brings strengths to the task, ensuring that the games are both engaging and educationally strong. There is also a body of literature relating to effective elements of commercial computer games and serious games for online learning. Links have been made between gaming and learning mechanics to emphasize instructional value [27]. We propose that the playful platform of computer gaming, the accessibility of the program online, the therapy and manner in which the therapy is delivered, and the way the content is structured to maximize learning all contribute to effective serious games for mental health.

By understanding how an intervention supports a user, we hypothesized we could identify serious game design elements that contribute to increased user engagement and adherence to therapy. We sought to do this by exploring user experiences with SPARX (smart, positive, active, realistic, X-factor thoughts), a self-help tool developed by authors of this paper (TF, ML, KS, MS) and others. SPARX was designed to deliver cCBT to adolescents aged 12 to 19 years old experiencing mild-to-moderate symptoms of depression using an engaging computerized platform.

**Figure 1:**
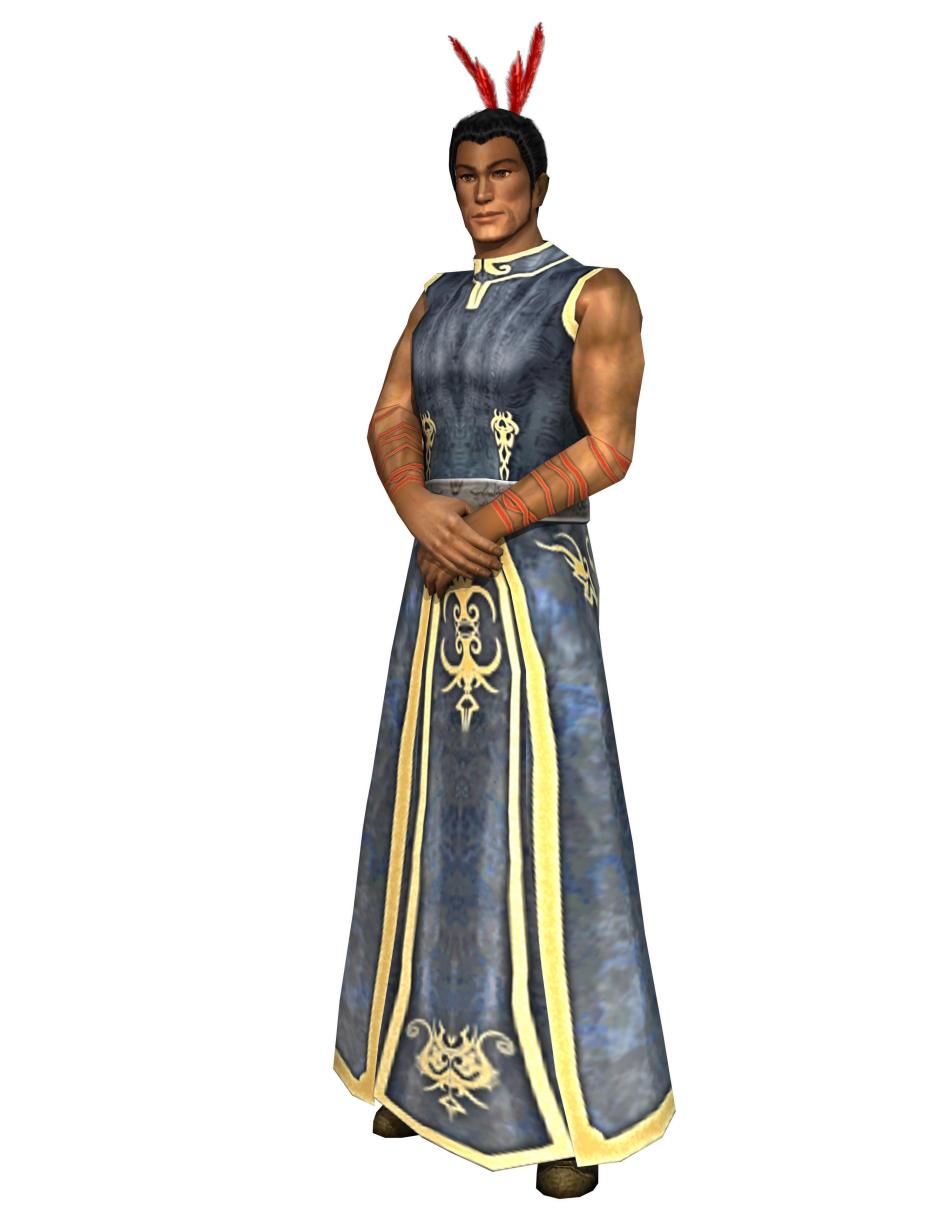
SPARX: the guide.

**Figure 2:**
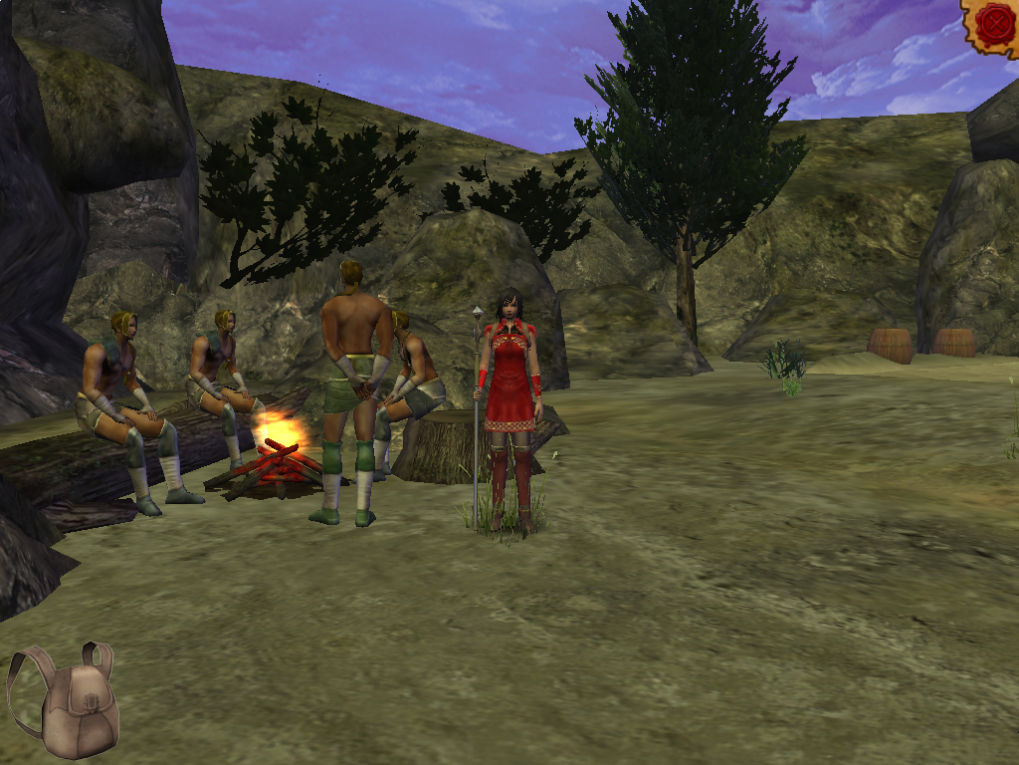
SPARX: canyon dwellers.

**Figure 3:**
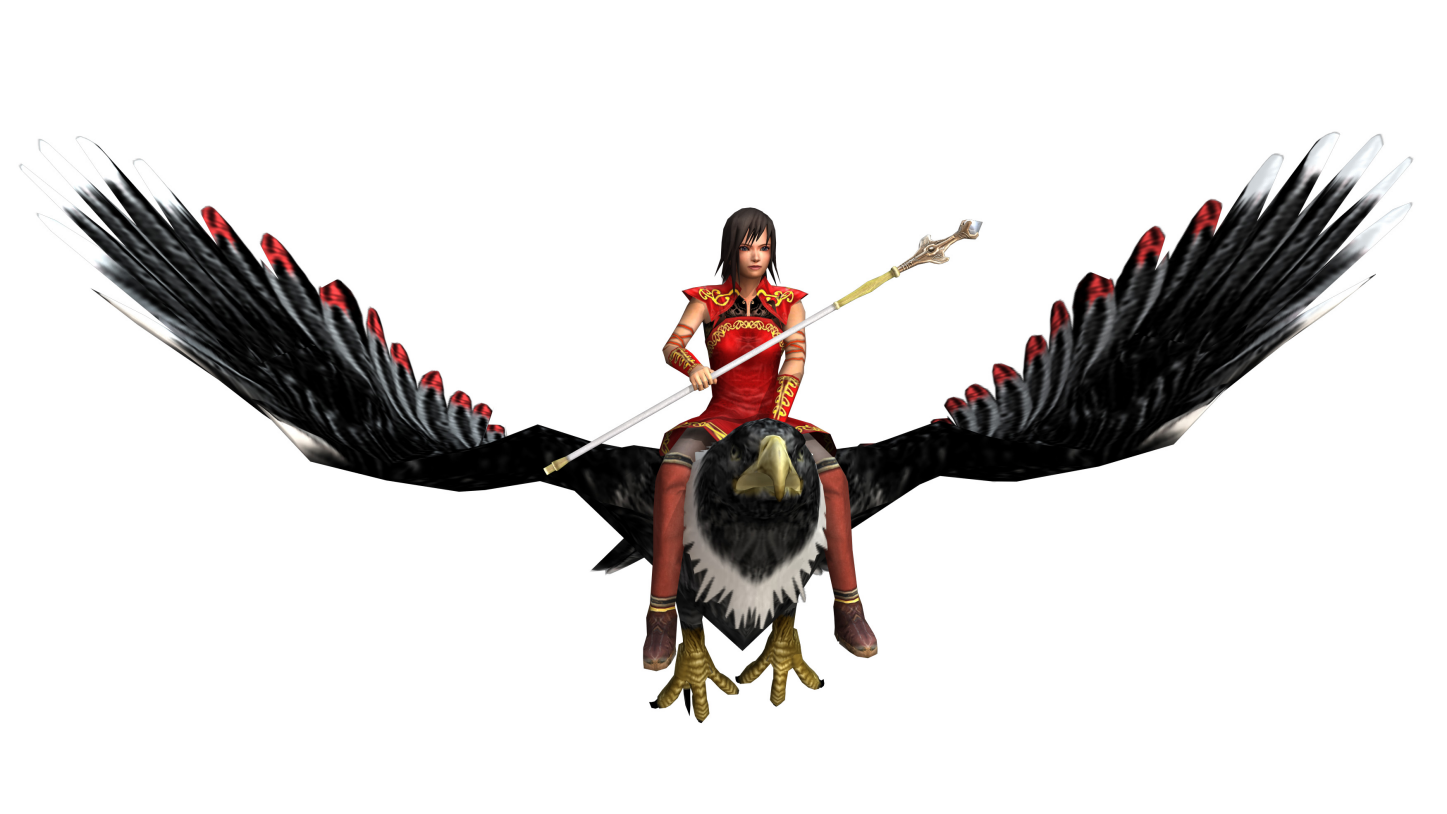
SPARX: user avatar flying on the eagle Te Hokioi.

The development and testing of SPARX has been described previously [28-32]. In brief, SPARX was developed using CBT and learning theory, with input on game design from youth and stakeholders. It uses a bicentric frame of reference [33]. In each module, users are explicitly introduced to therapeutic content using a virtual therapist or guide (Figure 1) and then transition to a fantasy setting to undertake CBT-based challenges and develop CBT-based skills within an overall narrative of restoring balance to the fantasy world (Figures 2 and 3). Following this exploratory learning, users return to the guide at the end of each level to reflect on the tasks and how they might be applied in their own lives. SPARX was shown to be at least as good as usual care (primarily counseling delivered face-to-face by a mental health clinician) for young people seeking help for low mood or depression [7]. It showed promising results in exploratory trials with Māori youth and students in alternative education programs, and a modified version (Rainbow SPARX) showed promise for sexual minority youth [29-31]. Youth trialing SPARX have also reported a high level of satisfaction and engagement with the program [28-32]. In the course of design and testing the program, a considerable amount of qualitative data was collected from these user-participants (Table 1). The user experiences are those of young people in community settings, thus targeted individuals in the pre-intentional to intentional stages according to HAPA. While data from these studies concerning young people’s satisfaction with the program and opportunities to improve it have been reported in previous publications, the data have not been combined across these different groups, and no systematic exploration of design elements has been undertaken.

In this study, we sought to define design elements from a user-centered perspective, specifically for youth aged 12 to 19 years, by extracting from all the SPARX focus group and interview data those features that users perceived as being most important in meeting their goals. We hypothesized important elements of the four areas—a playful platform of computer gaming, the accessibility of the program online, the therapy and manner in which the therapy is delivered, and the way the content is structured to maximize learning—could be identified within SPARX and linked by the results with evidence of supporting the user experience. This analysis is useful because serious games for mental health are seldom described in depth and there is little research to elucidate components of serious games that might be useful or appealing. Articulation of a framework of critical design elements could facilitate theory development and testing in this new field.

##  Methods

### Qualitative Data

Approval for this study was granted by the Tasmanian Social Sciences Higher Research Ethics Committee in Australia (H14061). The qualitative data used were gathered during five separate studies of five different user groups to explore the acceptability of SPARX. The selection of participants, approval and consent processes, methods of data collection, and transcription of the interviews are described in the respective papers for which the data were gathered [28-32].

All checked and corrected interview and focus group transcripts from these studies were used in this analysis.

The interviews were conducted with participants from groups that had three different experiences of the SPARX program: youth and family members, practitioners, and community elders during the program design stages (design); youth shown components of the program once it had been finalized (preview); and youth who had used the finalized program as part of a formal research trial (users). Each brought a particular perspective and experience, constituting a form of triangulation (Table 1).

**Table 1:** Interview participant data.

Number of groups or interviews		N	Age range (years)	Characteristics	Participant code
**Design—viewed a prototype**					
	6 groups, 1 interview	26	16-18	19 Māori 16-18, 7 parents/caregivers (15 female, 11 male) MHS NA^a^	Māori 1-26
	3 groups	10^b^	16-27	Lesbian, gay and bisexual young people (5 female, 4 male) MHS NA^a^	Rainbow 1-9
**Preview—viewed or tested one module**					
	4 groups, 1 interview	16	13-18	Rural Australian youth (4 female, 12 female) MHS NA^a^	Aus 1-16
	9 groups	39	13-16	Youth excluded from mainstream education (10 female, 29 male) MHS NA^a^	AE 1-39
**Users—participated in a trial/program**					
	25 interviews	25	13-19	Young people attracted to the same sex, both sexes, or not sure (13 female, 12 male) MHS NA^a^	Rainbow (User) 1-25
	39 interviews	39	13-16	Youth excluded from mainstream education with symptoms of possible depression using the Children’s Depression Rating Scale Revised (CDRS-R), 9 without symptoms. (15 female, 24 male).	AE (User) 1-39
	5 interviews	5	14-16	Youth attending mainstream school presenting with symptoms of mild-moderate depression using CDRS-R. (4 female, 1 male)	Māori (User) 1-5

^a^Mental health status not assessed in sample.

^b^One participant participated in two focus groups (ie, there are nine unique individuals).

Full transcripts of all interviews and focus groups were obtained and were subjected to iterative thematic analysis using NVivo version 10 software (QSR International) for organization. This study proceeded in two main stages: a hypothetical model was derived from the literature for the purpose of testing against the available interview data and confirmatory testing of the model was performed through the use of qualitative data.

### Creation of the Model

The data and publications describing the views of participants of SPARX were initially reviewed to assimilate emerging themes. From this an existing theoretical model of behavior change was selected which was relevant to the psychological constructs being described in the data. We then sought theories and features which pertained to the four areas: playful platform of computer gaming, the accessibility of the program online, the therapy and manner in which the therapy is delivered, and the way the content is structured to maximize learning.

### Confirmatory Testing of the Model Through the Use of Qualitative Data

#### Coding User Perceptions

An initial coding tree was established using the psychological constructs of the selected continuum model. Perceptions that did not fit within these constructs were coded as divergent themes. Initial coding was conducted by one member of the research team (CC) and validated at frequent intervals by a second member of the research team (HB). Neither of these authors was involved in the development of SPARX. Codes were then checked and refined with three other members of the research team (TF, KS, ML). Perceptions did not always neatly fit within one construct. Where a block of text appeared relevant to two constructs it was coded to both. The focus was not to quantify views relating to each construct but to capture important perceptions of the game according to participants. Within each construct, subnodes were established to organize perceptions into the four proposed design areas. This was either directly observable in the information or latent in the underlying experience.

#### Mapping User Perceptions to Design Elements

Within each construct, similar perceptions were then grouped into a common thread and entered into an Excel (2010) spreadsheet. The common threads were then mapped to the design element which was most explicit in the user perception.

## Results

### Creation of the Model

Themes reported in the various qualitative studies showed users valued the choices and control SPARX offered; the game was accessible to them when they wanted it, it protected their privacy, and it existed in a medium with which they were familiar. Engagement with the program arose from the playful medium, customizing their own character and, for those users who had completed the program, the sense they had benefited from the program and that the characters cared about them and gave them hope.

### Psychological Constructs

SDT proposes that when people perceive they have more control over their treatment, a sense of competence in the activities and tasks required of them, and a sense of being cared for and connected with another, they will be more likely to integrate learning and behavior change [22]. SDT has been applied to psychotherapy, education, online learning, human motivation, and health [23-29]. The three constructs, autonomy, competence, and relatedness, are seen as central to an individual’s sense of self and well-being and key motivators toward changing behavior. Supporting strategies such as positive regard, feedback, and structure facilitate motivation [34-40]. These three constructs also align well with important features in HAPA—self-efficacy (having confidence in performing tasks), social resources, and risk aversion.

As an established theory incorporating a motivational basis for effective change, SDT was likely to be a useful tool in understanding the perspectives of users and explaining the importance of supporting these with appropriate game design.

### Serious Game Design Elements

#### Overview

The theories pertaining to the four areas we proposed as contributing to serious games were selected for their relevance to the strategies that were adopted intentionally by the developers of SPARX and to classification of potential design elements (Table 2).

#### Elements Contributing to Enjoyable Computer Games

Computer game play is a worldwide phenomenon with an increasingly diverse participant base. Players are engaged using a mix of rich graphics and audio to undertake challenging quests or explore alternative worlds. The key design features that influence player enjoyment have been explored [41,42], and taxonomy of six design elements has been proposed and empirically tested [43,44].

#### Elements Relating to the Accessibility of Online Content

In the 1990s, the World Wide Web Consortium launched the Web Accessibility Initiative to advise web builders on strategies to enable equitable access to web sites. The Web Content Accessibility Guidelines 2.0 are the current standard and emphasizes testable principles embracing dynamic, rich environments to ensure Web content is more accessible [45]. While specifically applicable to web content, it provides a useful standard for design or evaluation of accessibility.

#### Elements of the Therapeutic Relationship

In examining the evidence for a link between a positive therapeutic alliance and effective outcomes for patients in community mental health services, Howgego and colleagues summarized historical development and contemporary theory constructs [46]. Bordin’s working alliance describes the relationship between a person seeking change and the change agent as one of the keys to the change process [47]. Fundamental to success is the active role clients play and the degree to which they value and believe in the purpose of the intervention and the process by which to attain change. While Bordin proposed generalizability of the working alliance model, it has been adopted predominantly in mental health, with a number of studies linking effective patient outcomes to a positive therapeutic alliance [46].

#### Elements Contributing To Learning Through Immersion

Existing theories of learning have been drawn upon to support the emerging pedagogy of online learning, supplemented with factors unique to this medium. Situated learning is a constructivist social learning theory [48] based on participatory knowledge acquisition from authentic contexts requiring activity, expert guidance, modeling of behavior, and a community of practice. Newcomers conduct simple, low-risk tasks, becoming familiar with language and organizing principles, and mature through a more active central role. Within an immersive interface, interaction of the participant’s avatar with other virtual characters can simulate a problem-solving community.

Immersion refers to the impression the user is participating in a real experience despite the user inherently knowing that some of the situations are not just unreal but impossible. Sensory, actional, and symbolic factors strengthen the degree of immersion [49]. Sensory immersion replicates digitally the experience of being in a three-dimensional space, using different camera angles, surround sound, motion, or vibration. Actional immersion allows the participant to initiate actions impossible in the real world (for example, flying on a bird), which intensifies the degree of user concentration. Symbolic immersion involves triggering powerful semantic psychological associations via the content of an experience.

Other ways which have been shown to enhance educational outcomes in an immersive environment are multiple perspectives and simulation of the real world [33]. Changing a participant’s view, or frame of reference, from the exocentric (providing an external view of an object or thing) to the egocentric (providing an internal view of the object or thing) strengthens actional immersion and motivation through embodied, concrete learning [33]. Transfer refers to the ability to conceptualize knowledge learned in a way that allows an individual to use it in practical, real-life environments. Simulated learning environments which reflect the real world are thought to aid in transfer of knowledge more than, for example, passive face-to-face counseling sessions or classroom activities.

**Table 2:** Design elements of computer game, accessibility, working alliance, and learning in immersion.

Area	Elements	Description
**Computer Game**		
	Challenge	Overcoming obstacles and challenges to master and beat games
	Companionship	Socializing and cooperating with others
	Exploration	Unfamiliar places, hidden things, different strategies, discovery
	Fantasy	Enjoyment of fantasy worlds, fictional characters, unreal abilities, imaginary creatures, role-playing
	Fidelity	Realistic 3D graphics, animation, sounds
**Accessibility**		
	Perceivable	Content is easy to see, hear; presented in different ways
	Operable and navigable	Function available from keyboard; users can find content and navigate easily; creates no well-being issues
	Understandable	Text is readable and understandable; game operates in predictable ways
	Robust and reliable	Interpretation clear, minimal errors, secure, compatible
**Working alliance**		
	Goal	The outcome the person values and perceives will improve their circumstances
	Tasks	A series of relevant and efficacious tasks, which the person perceives will assist in attaining the goal
	Bond	Positive personal attachments such as trustworthiness, confidence, expertness, attractiveness, acceptance, empathy, nonjudgmental, and sincerity
**Learning in** **immersion**		
	Activity	Through tasks, puzzles, movement, and feedback
	Expert guidance	Builds on and adjusts existing knowledge
	Modeling of behavior	Behaviors learned by observation and modeling
	Community of practice	Where newcomers conduct simple, low-risk tasks, becoming familiar with language and organizing
	Sensory, actional, and symbolic factors	Replicates the experience of being in a 3D space; participant able to initiate actions; content triggers semantic responses
	Multiple perspectives	Changing a participant’s view of an object from external to internal
	Simulation of the real world	Aids transfer of knowledge from conceptual to real-life

### Confirmatory Testing of the Model Using Qualitative Data

#### Coding User Perceptions

User perceptions mapped well to the broad constructs of autonomy, competence, and relatedness and to the four groups of computer game, accessibility, working alliance, and learning in immersion. Overall, the content across the participant groups and interviews was analogous with very similar quotes evident in the data, suggesting both a level of saturation and a high level of agreement regarding important design elements.

#### Perceptions of Autonomy

All groups appreciated accessing a recommended treatment without having to be referred to a counselor or other mental health service. Reasons varied from the cost or unavailability of services, stigma associated with getting help for mental health issues, having to tell someone else about problems, not having the confidence to talk about things, not being able to make sense of or find words to describe their feelings, or feeling embarrassed having to tell others. Users liked being able to talk or reveal their feelings to the computer without fear of being judged as well as learning skills and activities they could choose from and apply in their own lives*.*

The utility of having SPARX on their own computer, in their own space to play at a time that was convenient for them was central to their sense of control and protecting their privacy. Options to choose and personalize a character or avatar allowed individuals to express themselves as someone other than their own actual identity in a world other than their own. Several participants did not favor a computer game to access help, preferring face-to-face counseling instead. Furthermore, some participants did not have ready access to a computer. Examples of mapped participant quotes are provided in Textbox 1.

**Example quotes of user perceptions of autonomy.**

Computer Game:

I think it is a fairly good idea to have it so that you can customize it. It is a way of expressing yourself.

Rainbow 2/9

It’s a sort of a Warcraft thing where you pick your characters and go into a whole different world. I love fantasy.

AE (User) 4/39

Accessibility:

It was good because I felt that I had the control in that it was something that I could just do by myself.

Researcher then asks,

How could you ensure that you had control over it?

Well, seeing as it was on the computer I could just put it in my own file and no one else would go there and they would leave it to just me. It was good.

Rainbow (User) 25/25

Working Alliance:

So you are learning it from a computer game but it is still a really recommended thing.

Rainbow (User) 3/25

They don’t really have to talk to an actual person about it, and that way they don’t have to worry about getting judged with the feedback and stuff like that.

Aus 2/16

You have more control [with SPARX compared to counselor]. You can’t just leave a counselor that you don’t like.

AE (User) 1/39

Learning in Immersion:

I wanted to do it this way. I wouldn’t have liked being told what to do.

AE (User) 2/39

#### Perceptions of Competence

Quests to unknown worlds to accomplish tasks and collect gems as rewards were perceived as fun. While a sense of accomplishment was reported when puzzles and challenges were completed, the level of challenge difficulty experienced by participants varied greatly—from too easy to too hard.

Ease of operation, predictability of controls and actions, and content that was easy to see and hear supported a feeling of competence. Conversely, users were critical and frustrated when, for example, content was incompatible with their web browser or operating system. Users valued and gave examples of learning skills they could and had used in real life, conferring a real sense of achievement. Modeling of behavior was evident in the way participants described their learning experiences using SPARX.

Observing game characters going through problems similar to their own and helping the characters to overcome these in the game externalized the problem for users and instilled confidence to try these strategies in the real world. Where participants found the challenges too easy they felt a loss of engagement and subsequently thought that the program should be for a younger audience. More interaction was suggested as a strategy to combat this. Examples of mapped participant quotes are provided in Textbox 2.

**Example quotes of user perceptions of competence.**

Computer Game:

Cass [a character in SPARX] was cool because I felt cool helping her, you felt good.

Rainbow (User) 16/25

Accessibility:

The one thing I really, really hated about it was—you know how when you go to move your character you have to click. I reckon it would be better if you could use the arrow keys.

Rainbow (User) 3/25

When I was in the Ice-land level and I spent five minutes looking for the Yeti and it was right in front of me but I didn’t notice it because it was the same sort of white as the rest.

Rainbow (User) 7/25

Working Alliance

It gives good advice. It actually gives you real life techniques and skills that you can use and that are easy to use. And you are able to use them in everyday life.

Māori (User) 2/5

I was not really depressed at the beginning—I was just really angry—but it was still useful, I am less angry now…I don’t hate.

The researcher then asked,

Is this different from before you did SPARX?

Yes…I used the take 10 seconds, walk away…it has changed me. My Dad is happy with the changes, he is proud of me changing.

AE (User) 6/39

I think the negotiating one to solve it or sort it, about how you have to think half-half kind of thing. I think that works really well in school. I have used it on my teacher.

Rainbow (User) 8/25

I haven’t had a fight since it started. I’m not getting into trouble since SPARX. I learn more now, concentrating in class. SPARX taught me confidence.

AE (User) 8/39

Learning in Immersion:

When the bird comes out of the box it always speaks in a nice way—doesn’t shout. You have to practice saying things in a good way.

AE (User) 7/39

#### Perceptions of Relatedness

The richness of the computer game interface was evident in comments from the participants. For instance, many people commented that the characters were likeable, the 3D graphics were appealing, and the fantasy-based program was valuable. Fun was expressed as a feature of computer games in general: the settings, adventures, quests, and different characters in the game. Users related to various and different fantasy characters in SPARX. Being able to represent abstract concepts as something real within a fantasy computer game genre was also perceived as enjoyable. Users identified preferential character qualities including attractiveness, expertise, empathy, warmth, and sincerity. Immersive factors helped to engage participants by making them feel like they were part of the program. It was acknowledged the program would not suit everyone. A small number of users reported the language was too simple and that, by extension, they considered the characters patronizing. Examples of mapped participant quotes are provided in Textbox 3.

**Example quotes of user perceptions of relatedness.**

Computer Game:

It is really cool. It is like a real life thing in an imaginary world so it is really fun and more engaging. Because if it had real people it would be a bit boring.

Māori (User) 2/5

I really liked Hope [a talking character in the form of a bird]. I thought that was a cool idea…that hope was something tangible.

Rainbow (User) 10/25

Accessibility:

I am not paying much attention to the words in the box for some reason. I think I am more of an audio person.

Māori 1/26

Working Alliance:

The bird is cute—Hope. Yes, that was my little favorite thing on SPARX—Hope.

The researcher then queried,

What was it about the bird of hope that really stuck with you? Because she said “I am always here to help you.” It was so cute and just the color of her. She looks so beautiful. I wish she was my pet.

Māori (User) 3/5

When he [the guide character] asks how I‘ve been—that was good. I like that someone cares.

AE 9/39

And it never said you were wrong, it just said maybe try another way or that sort of thing.

The researcher then queried,

Why do you think that was important?

Well, if people keep saying you are wrong, you are going to give up.

Rainbow (User) 7/25

I thought one of them was good looking plus the voice over was…but, no, it is a game.

Rainbow (User) 20/25

Learning in Immersion:

You know the Gnats [Gloomy Negative Automatic Thoughts]? They sounded like Voldemort which I thought was helpful because they are evil.

Rainbow (User) 1/25

It was cool. And you can feel as if you are in the actual game itself.

The researcher then queried,

How would you describe that process of being in it?

Well, with the guide you felt like he was talking to you and not to a character as other games do. And you are controlling the person—walking by itself and that type of thing.

Māori (User) 2/

#### Mapping User Perceptions to Design Elements

Similar user perceptions were then grouped. For example, comments relating to how participants could use the program independently had been linked to the SDT construct autonomy, and where these were a function of the utility of the program, they were organized into the subnode accessibility. Similar perceptions of this were grouped into the thread “I can use the program where I want, when I want, how I want.”

While the perceptions most explicitly identified features of the serious game from within the group to which the perceiving thread was organized, at times other design elements were latent in user perceptions. For example, users perceived gaining skills, based on CBT tasks, that they could use in real life. Those user perceptions were linked to the competence construct and organized within the working alliance group. The supporting features of the serious game most evident were the practical CBT skills based within the design element of tasks of the working alliance. While this mapping is most obvious in the user perception, to teach this skill the game drew upon other elements which were less explicit in the participant data. These included working alliance factors (encouragement and feedback) learning pedagogy (initial learning of deep breathing, expert guidance about when and how to use it, modeling the activity, opportunity to practice the skill), multiple perspectives (virtual therapist) and immersive factors (actional and sensory); computer gaming (fantasy world characters, realistic animation, graphics, and sound); and accessibility (clear, perceivable content presented in both audio and text format, available widely through online or portable media). These elements were noted but not mapped directly to that perceiving thread.

**Figure 4:**
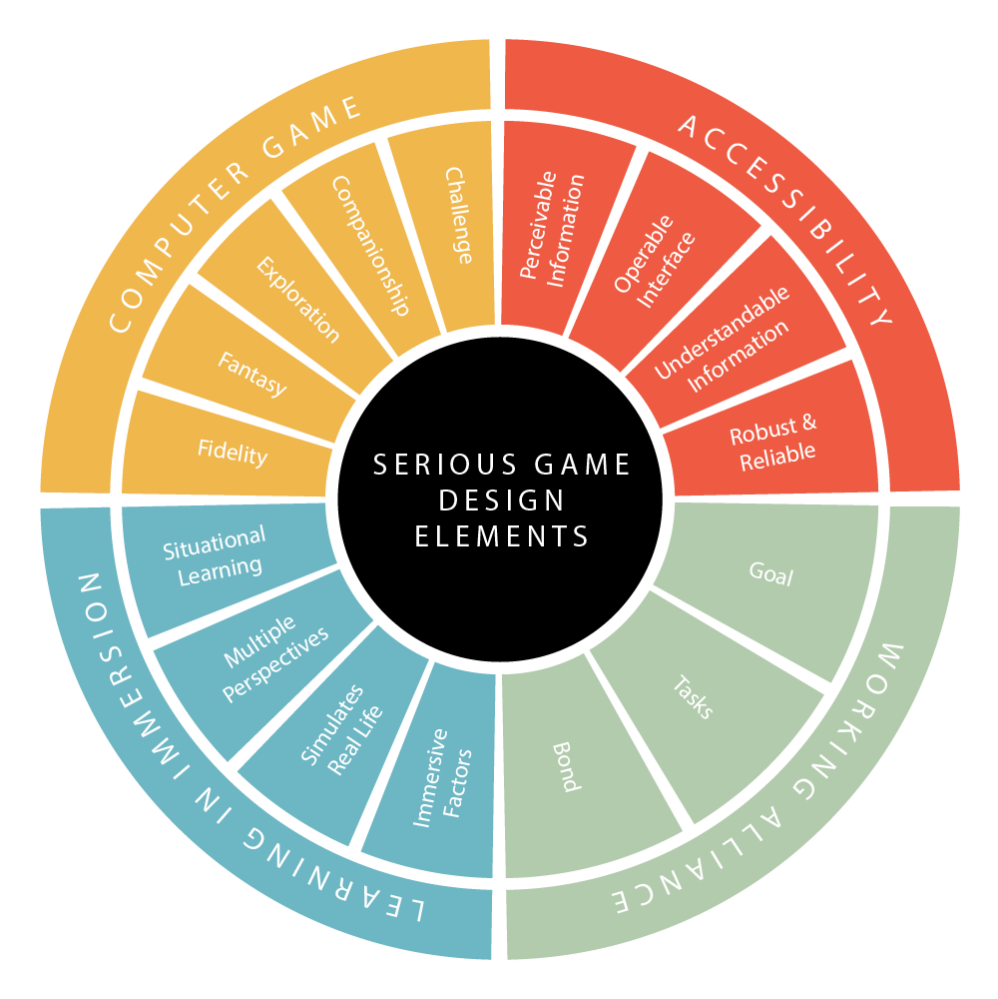
Serious game design elements.

**Figure 5:**
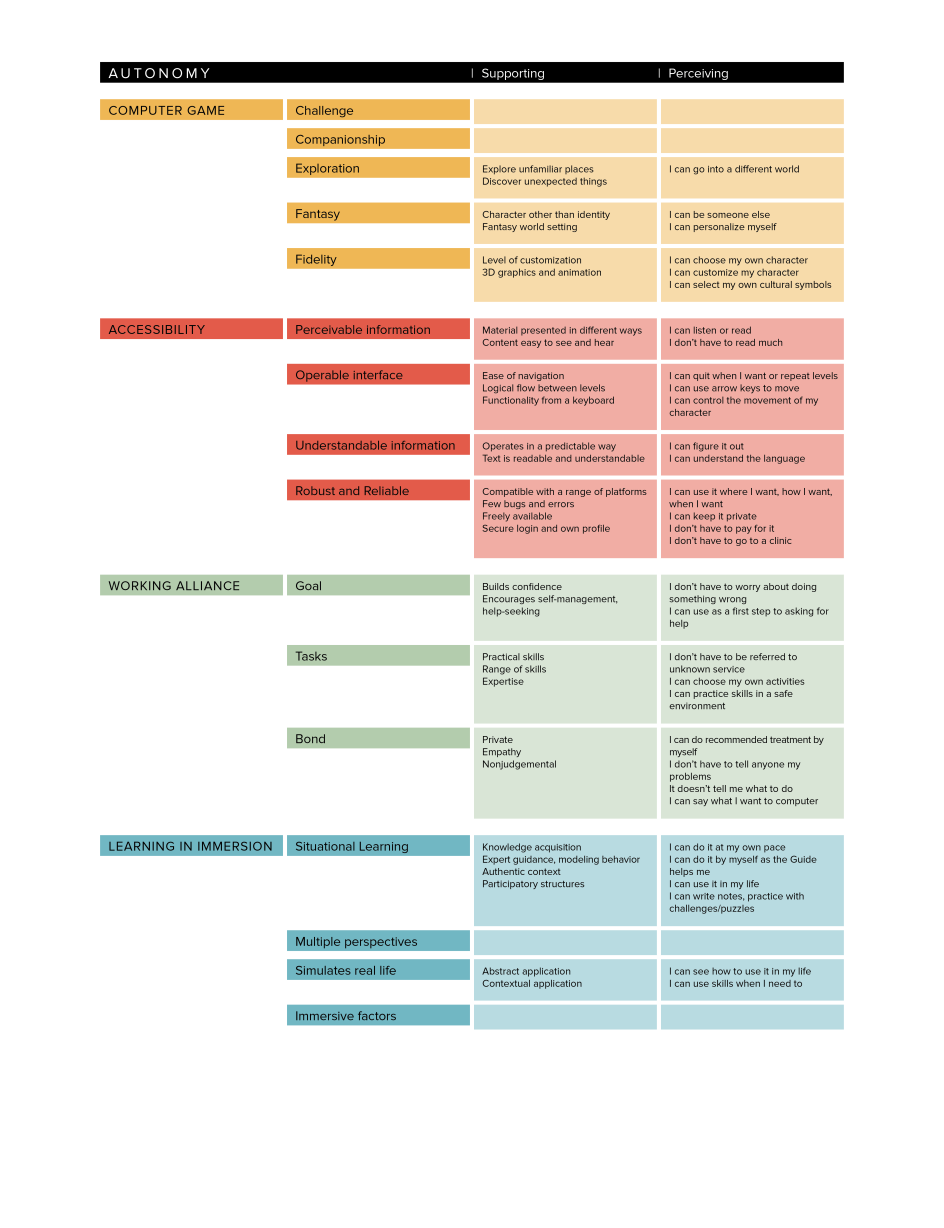
User perceptions of autonomy mapped to supportive features of a serious game.

**Figure 6:**
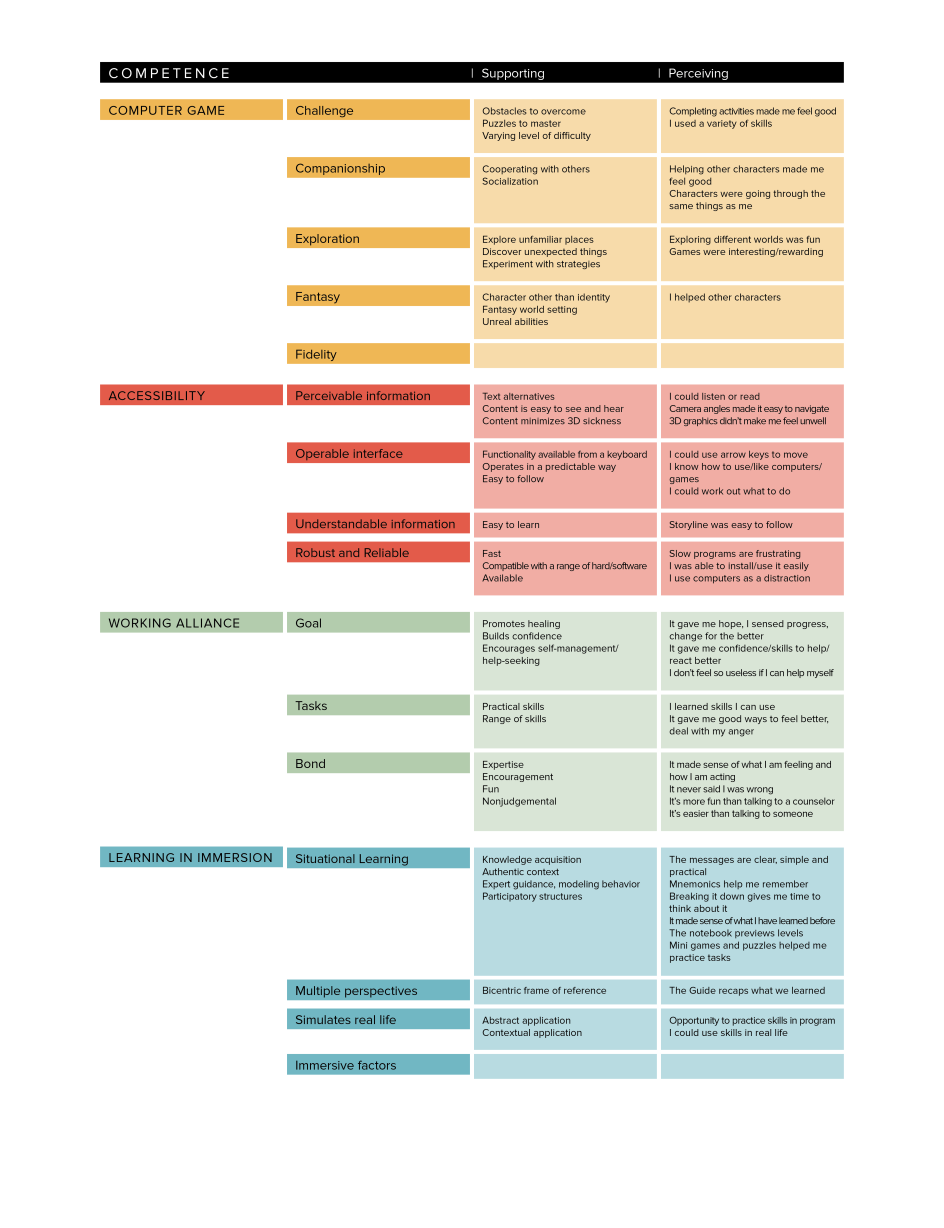
User perceptions of competence mapped to supportive features of a serious game.

**Figure 7:**
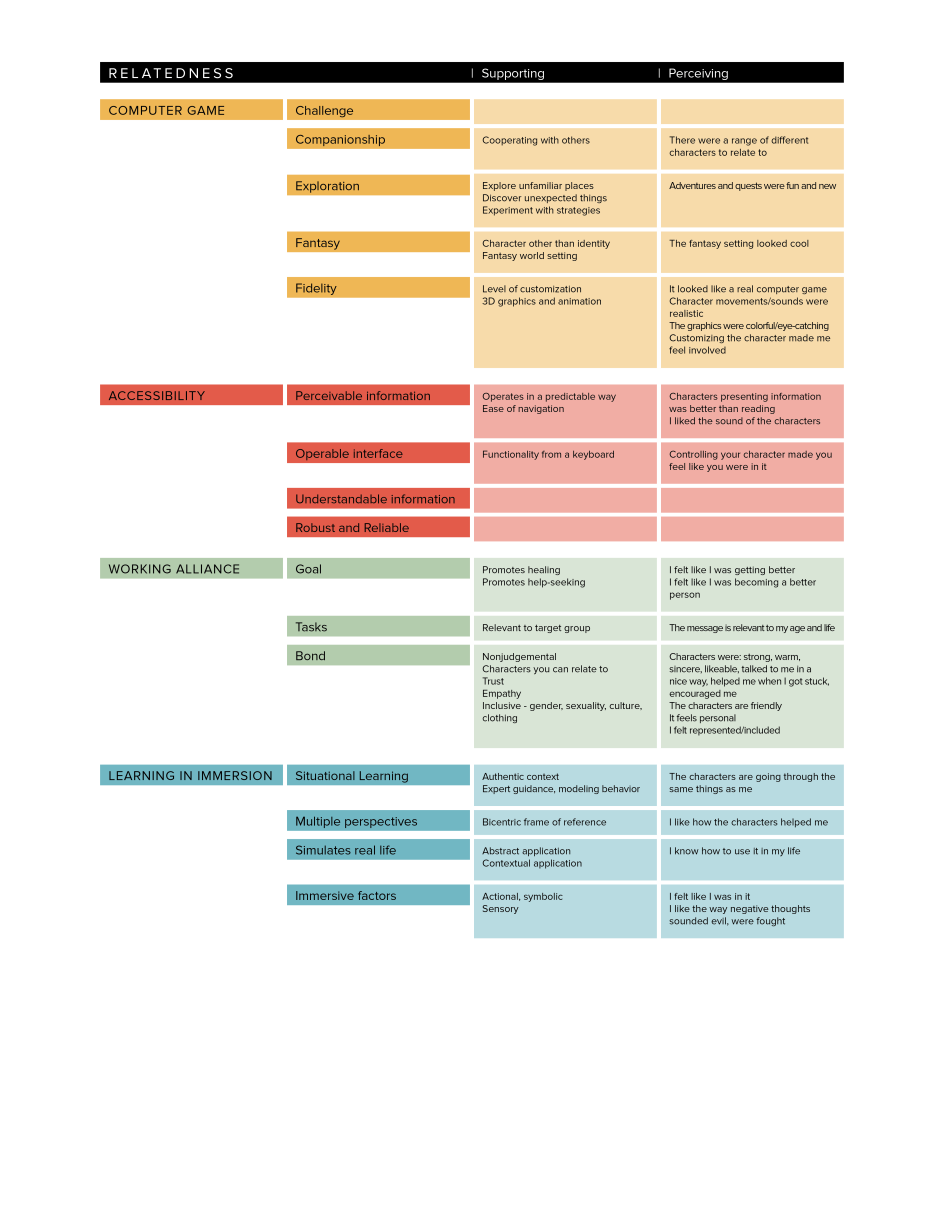
User perceptions of relatedness mapped to supportive features of a serious game.

We present the model of design elements in Figure 4, with the groups computer game, accessibility, competence, and relatedness color-coded orange, red, green, and blue, respectively. The design elements are presented for autonomy (Figure 5), competence (Figure 6) and relatedness (Figure 7) with the four groups color-coded to correspond to the main diagram (Figure 4) for interpretation. The perceiving threads are those expressed by users in the data. The supporting features are those identified in the four groups in the model and found in the game. The design elements that are not mapped directly to any user perceptions in that SDT construct were noted as supporting but were more latent in user perceptions such as those described in the CBT task above.

##  Discussion

### Self-Determination Theory and Serious Game Design

The serious game designer faces a complex task in marrying gaming elements with therapeutic and learning goals without compromising either. The groups of design elements shown in Figure 4 are strongly supported by and map well against the user perceptions outlined in our findings and represent a potentially useful tool for serious game designers. By further mapping these elements against the constructs of SDT (Figures 5-7), we provide a theoretical dimension to the model that opens up promising avenues for future exploration.

A major problem facing serious game designers is that while users have strong ideas about what will work for them, they are generally unable to consciously synthesize and/or articulate these requirements for the designer. The framework and model presented in this paper articulate the context of user needs and will assist developers to bridge the cultural divide and resolve some of the tensions between themselves and the therapy and pedagogy experts whose input will be vital in designing a serious game that meets therapeutic and learning goals. Articulating young people’s views of the game SPARX and matching these to supportive design features to improve the quality of community-based mental health interventions is important as over 75% of mental disorders commence before the age of 25 years [50].

HAPA distinguishes between the processes that motivate people to change (pre-intentional stage) and the processes that lead to the actual health behavior (volition stages) [25]. During the first stage, self-efficacy, outcome expectancies and risk perception affect motivation. During the second stage, people need detailed instruction on how to perform the desired action, and they must be confident that they can accomplish this. Thus, the constructs of SDT are important considerations at these stages of health intervention. The SDT framework presented contextualizes how young people perceived these factors and the serious game design elements which supported them.

Young people want help for mental health issues, but our findings reveal that there is also a strong need to be able to control how they access services or get help. This sense of autonomy at the pre-intentional stage was engendered by having recommended therapy available to them without having to be referred to or attend a clinic, having a program on their computer so they could use it where and when they wanted, having choices to personalize their avatar, and being able to select activities which were relevant to their life. These preferences regarding choice and control parallel normal developmental trajectories of exploring independence, autonomy, and identity during adolescence [51]. Given the transformative opportunity adolescence presents in biological, psychological, and social domains [52], matching any appropriate mental health intervention to the developmental concerns of this phase is crucial for positive treatment outcomes.

A sense of competence was supported by design elements from all four groups. The efficacy of the tasks and the extent to which they made the individual feel like they were improving was evident in powerful perceptions in the data of healing and gaining confidence. These facets of the working alliance seemed to be the strongest contributors to a sense of competence, yet more latent in perceptions was the deliberate application of learning pedagogy to the organization and presentation of content to impart knowledge in a more meaningful way. The exocentric virtual therapist provided observer perception and reflection, fostering more abstract, symbolic insights to help the user separate the problem from the person. An egocentric game component, by way of accomplishing a series of user tasks, enabled participants’ actional immersion and motivation through embodied, concrete learning [33]. Since most people have a sense of what makes a consistent fantasy world, players already have a level of fantasy world competence, while developers have a ready platform on which to build [53]. In SPARX, fantasy was an important tool: users valued externalizing their identity and enjoyed the unreal abilities of characters. The genre enabled developers to represent abstract concepts as concrete entities and participants to practice skills using immersive factors and perspective, which have been found to enhance educational outcomes [49]. Employing the most effective ways for users to navigate and control their interactions and movements are all important aspects of accessibility. The deliberate combination of strategies within the playful medium enriched the experience for the user and is likely to contribute to motivation at both the pre-intentional and volition stages.

Nevertheless, users had diverse views about the level of challenge within the game, implying it is unlikely one serious game will suit all and personalization may be needed. This aligns with the principles of HAPA, where predictors operate differently on those in one stage group compared with those in an adjacent stage group [25].

Relatedness support is not specifically addressed in the studies of SDT and online education; Sorebo and Haehre found no association between perceived relatedness and students’ levels of intrinsic motivation in educational computer games [37]. As issues of attrition challenge the efficacy of online therapies for mental health, incorporation of therapist support or active therapy ingredients is recommended [4,53,54]. Social support is considered a resource applicable at all stages in HAPA; lack of it can be a barrier to adopt or maintain health behaviors [25], and this relationship between the individual and the change agent is emphasized in Bordin’s working alliance [47]. The SPARX program was designed to be used independent of therapists but incorporated a guide as a virtual therapist. The text was chosen carefully to be therapeutic; the image of a powerful and supportive character (Figure 1) and an actor with a warm encouraging voice were specifically selected for the guide. User perceptions that the guide and other characters made them feel supported and cared for suggests that relatedness support can be achieved in serious games and it is perceived as helpful by users.

Whether serious games maintain the positive effects thus far described in efficacy studies has yet to be determined as most of the peer-reviewed literature describes open or randomized controlled studies where a short window of engagement exists with participants. Incorporating ways to get further help with a positive user experience (one in which the goal, sense of control, ability, and healing are valued) might promote further help-seeking behavior.

### Opportunities for Further Research

Users compared the experience of the serious game with commercial computer games. This may be an unrealistic expectation for serious game developers given the differences in development funding and the business models supporting the use of commercial games. While computer game users play for fun, serious games for mental well-being are targeting a specific and personal health-related goal. If the serious game sufficiently motivates the user to work toward a valued goal, the reliance on the serious game to entertain might be tempered. How the element of fun mediates user motivation is unknown in this context at this time. Tools to measure motivation have been used in online learning [34]. Measuring user motivation toward various serious games for mental health could assist in validating the critical design elements and further inform development.

It is likely that the relative importance of design elements will change as the goals of treatment, the target user audience, and the way in which a serious game is implemented vary. For example, we found participants in these groups and interviews were very keen to ensure their privacy, while Lederman and colleagues describe participants who had suffered psychosis valuing the peer support offered by an online social therapy tool [55]. This emphasizes the importance of consulting with potential users of the program during analysis and design.

### Limitations

This study examines the user perceptions of youth predominantly aged 12 to 19 years for one program, SPARX. The gaming elements among serious games for mental health issues differ. While the theories selected to support the qualitative data for this study were also selected for their generalizability, it is not presumed this model will be generalizable to other age groups, populations, or interventions. While once the domain of young people, computer gaming is gaining popularity among broader age groups and different cultures (in 2014, the average age of an Australian computer gamer was 32 years; 47% are female and 19% are older than 51 years [56]). It will be interesting to test this model against other age ranges and populations.

The divergent themes reinforce that this serious game does not suit everyone. Developing interventions which explicitly target one or more stages of HAPA may help us understand whether interventions engage people at one stage rather than another, whether the medium of gaming suits everyone, or whether a different set of constructs applies to their requirements. It was also clear the level of challenge varied among participants, and when tasks or language were considered too difficult or too easy, there was a loss of engagement. Cognitive capacity is thought to moderate the impact of treatment approaches, but it is unclear whether this is a factor of the cognitive capacity of the user or the way content is presented [49]. Some people would rather get treatment via traditional modes of therapy, specifically face-to-face therapy; others may not have ready access to a computer or the Internet. In general, young people who identified as gamers indicated a higher level of engagement with the concept of SPARX; however, it is currently unclear how important the design features are in engaging young people who don’t normally play computer games or who are reluctant to engage in any mode of mental health intervention or support. The focus group and interview participants represented some of the people most underserved by mental health services in the community. While their perspectives are unique, they may not be representative of the overall adolescent population.

Given four of the authors were codevelopers of SPARX, the risk of bias is inherent. The remaining authors were independent of the development of SPARX; members of this group conducted the initial thematic analysis and validation of codes.

### Conclusions

Mental health issues affect a large number of people, many of whom will not access care through traditional models of care. Serious games offer a means of extending the reach of evidence-based early intervention, but they need to be well designed to deliver therapy in a way that engages users and helps them. The methods used in this study allowed articulation of design elements from a user-centered perspective in a structured framework. The framework and model may provide a guide for developers to ensure programs support important user-centered requirements. The relative importance of the various design elements is likely to vary with the purpose of the serious game and goals of treatment. Involving users in development is imperative if serious games are to be fit for purpose.

## Appendix

Multimedia Appendix 1 Table 2: Interview Participant data-extended.

## Abbreviations

CBT: cognitive behavioral therapy
cCBT: computerized cognitive behavioral therapy
HAPA: health action process approach
SDT: self-determination theory
SPARX: smart, positive, active, realistic, X-factor thoughts

## References

[1] World Health Organisation (2013). Mental health action plan 2013-2020. Mental health action plan. In.

[2] National Institute for Clinical Excellence (NICE) Clinical guidelines CG90.

[3] Calear Alison L, Christensen Helen (2010). Review of internet-based prevention and treatment programs for anxiety and depression in children and adolescents. Med J Aust.

[4] Richards Derek, Richardson Thomas (2012). Computer-based psychological treatments for depression: a systematic review and meta-analysis. Clin Psychol Rev.

[5] Melville Katherine M, Casey Leanne M, Kavanagh David J (2010). Dropout from Internet-based treatment for psychological disorders. Br J Clin Psychol.

[6] Coyle David, Doherty Gavin, Sharry John (2009). An evaluation of a solution focused computer game in adolescent interventions. Clin Child Psychol Psychiatry.

[7] Merry SN, Stasiak K, Shepherd M, Frampton C, Fleming T, Lucassen MFG (2012). The effectiveness of SPARX, a computerised self help intervention for adolescents seeking help for depression: randomised controlled non-inferiority trial. BMJ.

[8] Khanna Muniya S, Kendall Philip C (2010). Computer-assisted cognitive behavioral therapy for child anxiety: results of a randomized clinical trial. J Consult Clin Psychol.

[9] Shandley Kerrie, Austin David, Klein Britt, Kyrios Michael (2010). An evaluation of 'Reach Out Central': an online gaming program for supporting the mental health of young people. Health Educ Res.

[10] Rizzo A, Newman B, Parsons T (2009). Development and clinical results from the virtual iraq exposure therapy application for PTSD. Virtual Rehabilitation International Conference.

[11] Fleming T, Cheek C, Merry S, Thabrew H, Bridgman H, Stasiak K, Shepherd M, Perry Y, Hetrick S (2015). Serious games for the treatment or prevention of depression: a systematic review. evista de Psicopatología y Psicología Clínica - Spanish Journal of Clinical Psychology.

[12] Dunn Dana S, Elliott Timothy R (2008). The Place and Promise of Theory in Rehabilitation Psychology. Rehabil Psychol.

[13] Noar SM, Benac CN, Harris MS (2007). Does tailoring matter? Meta-analytic review of tailored print health behavior change interventions. Psychol Bull.

[14] Limerick H, Coyle D, Moore J (2014). The experience of agency in human-computer interactions: A review. Front Hum Neurosci.

[15] Knowles SE, Toms SG (2014). Qualitative Meta-Synthesis of User Experience of Computerised Therapy for Depression and Anxiety. PLoS one.

[16] Cayton H (2006). The flat-pack patient? Creating health together. Patient Educ Couns.

[17] Detmer DE (2003). Building the national health information infrastructure for personal health, health care services, public health, and research. BMC Med Inform Decis Mak.

[18] Hesse BW, Hansen D, Finholt T, Munson S, Kellogg W, Thomas JC (2010). Social Participation in Health 2.0. Computer (Long Beach Calif).

[19] Ryan RM, Deci EL (2000). Self-determination theory and the facilitation of intrinsic motivation, social development, and well-being. Am Psychol.

[20] Deci E, Ryan R (2008). Self-determination theory: A macrotheory of human motivation, development, and health. Canadian Psychology/Psychologie canadienne.

[21] Bandura A (2004). Health promotion by social cognitive means. Health Educ Behav.

[22] Rogers RW (2010). A protection motivation theory of fear appeals and attitude change. J Psychol.

[23] Schwarzer R, Lippke S, Luszczynska A (2011). Mechanisms of health behavior change in persons with chronic illness or disability: the Health Action Process Approach (HAPA). Rehabil Psychol.

[24] Prochaska J, Velicer WF (1997). The transtheoretical model of health behavior change. Am J Health Promotion.

[25] Schwarzer R (1992). Self-efficacy in the adoption and maintenance of health behaviors: Theoretical approaches and a new model. Self-Efficacy: Thought Control Of Action.

[26] Marne B, Wisdom J, Huynh-Kim-Bang B, Labat JM (2012). A design pattern library for mutual understanding and cooperation in serious game design. Intelligent tutoring systems.

[27] Arnab S, Lim T, Carvalho M, Bellotti F, de FS, Louchart S, Suttie N, Berta R, De GA (2014). Mapping learning and game mechanics for serious games analysis. Brit J Educ Technol.

[28] Fleming T, Lucassen M, Stasiak K, Shepherd M, Merry S (2015). The impact and utility of computerised therapy for educationally alienated teenagers: The views of adolescents who participated in an alternative education-based trial. Clin Psychol.

[29] Shepherd M, Fleming T, Lucassen M, Stasiak K, Lambie I (2015). The design and relevance of a computerised therapy program for indigenous Maori adolescents. JMIR Serious Games.

[30] Lucassen MFG, Hatcher S, Stasiak K, Fleming T, Shepherd M, Merry SN (2014). The views of lesbian, gay and bisexual youth regarding computerised self-help for depression: An exploratory study. Advances in Mental Health.

[31] Fleming TM, Dixon RS, Merry SN (2014). ‘It’s mean!’ The views of young people alienated from mainstream education on depression, help seeking and computerised therapy. Advances in Mental Health.

[32] Cheek C, Bridgman H, Fleming T, Cummings E, Ellis L, Lucassen MFG, Shepherd M, Skinner T (2014). Views of young people in rural Australia on SPARX, a fantasy world developed for New Zealand youth with depression. JMIR Serious Games.

[33] Dede C (2009). Immersive interfaces for engagement and learning. Science.

[34] Chen KC, Jang SJ (2010). Motivation in online learning: Testing a model of self-determination theory. Comput Hum Behav.

[35] Standage M, Duda JL, Ntoumanis N (2005). A test of self-determination theory in school physical education. Br J Educ Psychol.

[36] Xie K, Debacker Tk, Ferguson C (2005). Extending the traditional classroom through online discussion: The role of student motivation. J Educ Comput Res.

[37] Sorebo O, Haehre R (2012). Investigating students' perceived discipline relevance subsequent to playing educational computer games: A personal interest and self-determination theory approach. Scandinavian J Educ Res.

[38] Ryan R, Deci E (2008). A self-determination theory approach to psychotherapy: The motivational basis for effective change. Canadian Psychology/Psychologie canadienne.

[39] Schoech D, Boyas J, Black B, Elias-Lambert N (2013). Gamification for Behavior Change: Lessons from Developing a Social, Multiuser, Web-Tablet Based Prevention Game for Youths. J Technol Hum Services.

[40] Vallerand R, Pelletier LG, Blais M, Briere N, Senecal C, Vallieres E (1992). The Academic Motivation Scale: A Measure of Intrinsic, Extrinsic, and Amotivation in Education. Educ Psychol Meas.

[41] Hunicke R, LeBlanc M, Zubek R (2004). MDA: A formal approach to game design and game research. Proceedings of the nineteenth national conference on Artificial Intelligence.

[42] King D, Delfabbro P, Griffiths M (2009). Video game structural characteristics: a new psychological taxonomy. Int J Ment Health Addiction.

[43] Quick J, Atkinson R, Lin L (2013). Confirming the taxonomy of video game enjoyment. Games, Learning, Society 8.0.

[44] Quick J, Atkinson R, Lin L (2012). Empirical taxonomies of gameplay enjoyment: Personality and video game preference. Int J Game-Based Learning.

[45] (2008). WCAG.

[46] Howgego IM, Yellowlees P, Owen C, Meldrum L, Dark F (2003). The therapeutic alliance: the key to effective patient outcome? A descriptive review of the evidence in community mental health case management. Aust N Z J Psychiatry.

[47] Bordin E (1979). The generalizability of the psychoanalytic concept of the working alliance.

[48] Lave J, Wenger E (1991). Situated learning: legitimate peripheral participation.

[49] Dede C (2008). Theoretical perspectives influencing the use of information technology in teachinglearning. International handbook of information technology in primary and secondary education.

[50] Hickie IB, McGorry PD (2007). Increased access to evidence-based primary mental health care: will the implementation match the rhetoric?. Med J Aust.

[51] MacLeod KB, Brownlie E (2014). Mental Health and Transitions from Adolescence to Emerging Adulthood: Developmental and Diversity Considerations. Canadian Journal of Community Mental Health.

[52] Weisz JR, Sandler IN, Durlak JA, Anton BS (2005). Promoting and protecting youth mental health through evidence-based prevention and treatment. Am Psychol.

[53] Ryan S, Slavatore K, Green J, Jongewaard D (2006). Online evolution: is there life beyond elves and dwarves?. Computer Gaming World 258.

[54] Spek V, Cuijpers P, Nyklícek I, Riper H, Keyzer J, Pop V (2007). Internet-based cognitive behavioural therapy for subthreshold depression in people over 50 years old: a randomized controlled clinical trial. Psychol Med.

[55] Lederman R, Wadley G, Gleeson J, Bendall S, Álvarez-Jiménez M (2014). Moderated online social therapy. ACM Trans. Comput.-Hum. Interact.

[56] Brand JE, Lorentz P, Mathew T (2014). Digital Australia.

