# Supplementary material for: Integrating Health Behavior Theory and Design Elements in Serious Games
Source: JMIR Ment Health. 2015 Apr 21;2(2):e11. doi: 10.2196/mental.4133 (PMC4607397; doi:10.2196/mental.4133)
Supplement: Multimedia Appendix 1 [file mental_v2i2e11_app1.pdf]

*Table 2 - Interview participant data - extended*

| Context | Focus groups or individual interviews | Number of groups or interviews | N | Age range | Characteristics of the participants | Participant Code |
|---|---|---|---|---|---|---|
| Design phase of SPARX (viewed a prototype level of SPARX) | Focus groups & Interview to explore perceived relevance of SPARX prototype among Maori and to help guide refinements for the final program | 6 groups 1 interview | 26 | 16-18+ | 19 Māori 16-18 year olds (including 4 teen mothers) and 7 Māori parents/caregivers of adolescents (Total of 15 female and 11 male participants). MHS NA[a] | Māori 1-26 |
| | Focus groups To explore perceived relevance of SPARX prototype among sexual minority young people and to helo guide refinements for a | 3 | 10[b] | 16-27 | Lesbian, gay and bisexual young people (5 female & 4 male) MH NA[a] | Rainbow 1-9 |

| | | | | | | |
|---|---|---|---|---|---|---|
| | Rainbow version | | | | | |
| Viewed or tested one module of SPARX | Focus groups & interviews To explore the acceptability of SPARX by youth in rural Australia and to explore whether and how young people would wish to access such a program. | 4 groups<br>1 interview | 16 | 13-18 | Young people from rural Australia (12 male & 4 female; 4 Aboriginal and 12 other Australians). MHS NA[a] | Aus 1-16 |
| | Focus groups To explore the acceptability of SPARX to young people in alternative high schools and to explore whether and how these young people | 9 groups | 39 | 13-16 | Youth 13-16 year olds (29 male, 10 female; 19 Māori, 15 Pacific Island, 5 New Zealand European or other) from alternative schooling programmes for adolescents excluded from mainstream education in New Zealand. MHS NA[a] | AE 1-39 |

| | | | | | | |
|---|---|---|---|---|---|---|
| | would wish to access such a program. | | | | | |
| Users of SPARX (participated in a trial and completed or had the opportunity to complete SPARX) | Interviews To explore perceived relevance and effectiveness of Rainbow SPARX among sexual minority young people in order to inform the development and dissemination of future programs with this group, | 25 interviews | 25 | 13-19 | Young people attracted to the same sex, both sexes or not sure of their attractions (12 male and 13 female) who had used Rainbow SPARX in New Zealand. 2 participants' sex assigned at birth differed from their gender identity (i.e. they were transgender). MHS NA[a] | Rainbow (User) 1-25 |
| | Interviews to explore perceived relevance and effectiveness of SPARX to young people in Alternative Education settings in | 39 interviews | 39 | 13-16 | Youth 13- 16 year olds in alternative education programmes for adolescents excluded from mainstream education (24 male; 15 Maori; 12 Pacific Island; 12 New Zealand European or other), 30 with symptoms of possible | AE (User) 1-39 |

| | | | | | | |
|---|---|---|---|---|---|---|
| | order to inform the development and dissemination of future programs with this group, | | | | depression using the Children's Depression Rating Scale Revised (CDRS-R), 9 without symptoms. | |
| | Interviews To explore perceived relevance and effectiveness of SPARX to Maori young people in order to inform the development and dissemination of future programs with this group, | 5 interviews | 5 | 14-16 | Young people 14-16 years of age (one male, 14) who were attending mainstream school. All Māori youth presented with symptoms of mild-moderate depression as assessed using the by (CDRS-R) | Māori (User)1-5 |
| Total | | 22 focus groups<br>66 interviews | 160 | | | |
